# Influenza vaccination patterns among at-risk patients during the Covid-19 pandemic—a retrospective cross-sectional study based on claims data

**DOI:** 10.1007/s15010-024-02175-3

**Published:** 2024-02-01

**Authors:** Andreas Plate, Christophe Bagnoud, Thomas Rosemann, Oliver Senn, Stefania Di Gangi

**Affiliations:** 1 Institute of Primary Care, University of Zurich and University Hospital Zurich, Pestalozzistrasse 24, 8091 Zurich, Switzerland; 2 Groupe Mutuel, Martigny, Switzerland

**Keywords:** Influenza, Vaccination, Population at risk, Covid-19, Pandemic, Switzerland

## Abstract

**Purpose:**

The Covid-19 pandemic may have encouraged at-risk patients to get vaccinated against influenza for the first time. As previous vaccinations are known predictors for further vaccinations, knowledge about individual vaccination patterns, especially in first time vaccinated patients, is of great interest. The aim of this study was to determine influenza vaccination uptake rate (VUR), individual vaccination patterns and factors associated with vaccination uptake among at-risk patients.

**Methods:**

The study design was retrospective cross-sectional. Based on claims data, VUR was determined for four influenza seasons (2018/2019—2021/2022). In a cohort subgroup, with data available for all seasons, VUR, vaccination patterns and factors associated with uptake were determined. At-risk patients were people aged ≥ 65 and adult patients with chronic diseases.

**Results:**

We included *n* = 238,461 patients in the cross-sectional analysis. Overall VUR ranged between 21.8% (2018/2019) and 29.1% (2020/2021). Cohort subgroup consisted of *n* = 138,526 patients. Within the cohort, 56% were never vaccinated and 11% were vaccinated in all seasons. 14.3% of previously unvaccinated patients were vaccinated for the first time in the first pandemic season (2020/2021 season). The strongest predictor for vaccination was history of vaccinations in all previous seasons (OR 56.20, 95%CI 53.62–58.90, p < 0.001).

**Conclusion:**

Influenza VUR increased during the Covid-19 pandemic, but only a minority of previously eligible but unvaccinated at-risk patients were vaccinated for the first time in the first pandemic season. Previous vaccinations are predictors for subsequent vaccinations and health care professionals should actively address at-risk patients’ vaccination history in order to recommend vaccination in future seasons.

**Supplementary Information:**

The online version contains supplementary material available at 10.1007/s15010-024-02175-3.

## Background

At the beginning of the Coronavirus disease 2019 (Covid-19) pandemic, public health authorities and healthcare providers expressed concern about the potential co-circulation of severe acute respiratory syndrome coronavirus 2 (SARS-CoV-2) and the influenza virus [1]. The co-circulation of both viruses would place more pressure on national healthcare systems [2], already under significant pressure. Seasonal influenza outbreaks are known to have a considerable impact [3–5]. From an individual patient perspective, evidence from the early phase of the pandemic suggested worse outcomes in patients with SARS-CoV-2 and influenza co-infection [6–8]. Therefore, to safeguard both the public healthcare system and patients from the preventable effects of influenza, national health authorities and various stakeholders have highly recommended influenza vaccination for the vulnerable population during the 2020/2021 winter season [1, 3, 9].

Influenza vaccination uptake rates (VUR) in numerous European countries are low [10]. In Switzerland, there has even been a decrease in influenza VUR in recent years [11]. Pandemics have the potential to impact influenza VUR [12]. Recent studies have shown an increase in influenza VUR during the Covid-19 pandemic. As previous influenza vaccination is a recognized predictor for vaccination in subsequent years [13, 14], identifying characteristics of patients who were vaccinated for the first time during the first pandemic season (2020/2021 season), specifically due to the pandemic, may support public health authorities and healthcare providers future actions to target these patients to encourage annual influenza vaccination. However, national surveillance carried out by the Federal Office of Public Health (FOPH) relies on cross-sectional data. As a result, information on individual patient history is not available, with only overall VUR reported [15].

Thus, the objective of our research was to determine influenza VUR among an at-risk population in Switzerland during the Covid-19 pandemic. Our focus was to identify individual vaccination patterns as well as patient-related factors associated with vaccination. Additionally, we aimed to identify characteristics of patients who received their first influenza vaccine during the first pandemic season when SARS-CoV-2 vaccination was not widely available. We supposed that during the pandemic, VUR increased and that patients with multiple risk factors were more likely to be vaccinated.

## Methods

Study design and database: The study design was retrospective cross-sectional. Groupe Mutuel (GM), a large Swiss health insurance company, provided anonymized data from mandatory health insurance [16]. In Switzerland, basic health insurance is compulsory for the entire population and covers the costs of all treatments after deductibles and co-payments have been met. This includes the costs of vaccinations that are recommended for the population according to the national vaccination plan. The influenza vaccination is recommended to all person groups at increased risks for complications. Health insurance companies must offer insurance coverage to all patients without restrictions. GM insured patients in all parts of Switzerland and covers about 12% of the Swiss population. There is no distinction between public and private outpatient care in Switzerland. According to the GM regulations, a random sample of 95% of identified patients was provided. However, data for subsequent influenza seasons were available as long as the patients were alive and insured by GM.

Participants: Our study included at-risk patients, namely individuals aged 65 or older and all adult patients (aged 18 or older) with a chronic condition eligible for influenza vaccination based on the current national vaccination schedule [17]: heart, liver, lung, cerebral and kidney diseases, diabetes mellitus and immunocompromised patients with autoimmune diseases, diseases requiring drug immunosuppression, human immunodeficiency viruses (HIV), cancer, lymphoma, leukemia or myeloma or transplantations. Participants were selected based on medication claims [18] and outpatient services [19]. Patients who received inpatient treatment during the observation period were identified using diagnosis codes [20] (Supplemental Table 1). We created a retrospective cohort for subgroup analysis by selecting all patients with data in each of the four influenza seasons. We divided the at-risk patients into two subgroups: the cohort subgroup and the remaining cross-sectional patient subgroup (CS).

Data description and definitions: Analyzed data covered four influenza seasons from 01/08/2018 to 31/07/2022: 2018/19 (pre-pandemic), 2019/20 (pandemic start), 2020/21 (first pandemic season, without widespread Covid-19 vaccination), and 2021/22 (second pandemic season, with Covid-19 vaccination available). The influenza season spanned from August to July in order to accurately assign individual claims to the clinical influenza season. WHO ATC codes (J07BB01, J07BB02, J07BB03, or J07BB04) were used to identify influenza vaccinations.

Patient characteristics were: age in years calculated at each season from year of birth, gender (male, female), long-term care: living in a nursing home (yes/no), Swiss citizenship (yes/no), health insurance model (family doctor, network or telemedicine call-center as gatekeeper; free choice model allowing free choice of physicians), deductible (level 1: ≤ 500 Swiss francs (CHF); level 2: 501–1500 CHF: level 3: 1501–2500 CHF), area of residence (defined according to the Swiss cantons [cantons represent the member states of Switzerland] main speaking language regions: French, German, Italian), number of all active pharmaceutical cost groups (PCG), surrogate for the number of comorbidities, number of general practitioners (GP) and specialists visits (continuous variable).

Outcomes: Our primary outcome was influenza VUR among at-risk patients during the Covid-19 pandemic. Secondary outcomes were the VUR among the different risk groups (patients ≥ 65 of age and/or patients with a chronic disease), and the rate of change in VUR between two subsequent seasons, e.g. season 1 and season 2: defined as (VUR in season 2-VUR in season 1)/(VUR in season 1). Finally, we aimed to determine VUR in the cohort subgroup, patterns of vaccination, and factors associated with vaccination uptake in the last season. Patterns were all combinations of binary status (N = not vaccinated; V = vaccinated) in each season, i.e. sequences of four elements. For example, N-N-N-N identified never vaccinated patients. Vaccination history, in the last season, was defined based on the first-three elements of the sequence as: no previous vaccination, one, two, three previous vaccinations. Finally, we aimed to describe the subgroup of first-time vaccinated patients (FTVP) in the first pandemic season (2020/2021), i.e. patterns N-N-V-N and N-N-V-V.

Statistics: Descriptive statistics were presented: for categorical or binary variables as number and percentage, N(%), and, for continuous variables, as mean (standard deviation (SD)) or median (interquartile range (IQR)) as appropriate. Available case analysis was performed. Comparisons, between the cohort subgroup and cross-sectional patients (CS) and between the FTVP patients and the others, were performed using chi-square test, for binary or categorical variables, and t-test or non-parametric Wilcoxon test, as appropriate, for continuous variables. Vaccination patterns for the cohort subgroup were presented using a waffle chart, i.e. a grid in which each cell represents a portion or percentage of the whole.

Univariable and multivariable logistic regression models were performed to identify factors associated with vaccination status in the last season. Variance inflation factor (VIF), generalized for logistic regression, was calculated for each predictor, using a criterion of VIF greater than 5 to detect multicollinearity. Only the cohort subgroup data was analyzed, since history of vaccination, together with all patient characteristics, was a predictor factor. Results were reported in tables with odds ratio (OR) and 95% confidence interval (CI). Test results were considered statistically significant at p ≤ 0.05, two-sided. All analyses and figures were performed using R version 4.1.0 [21].

The study was reported according to the STROBE checklist for cross-sectional studies.

## Results

We examined data of *n* = 238,461 patients in the cross-sectional analysis. Baseline characteristics are presented in Table 1 and Supplemental Table 2. In the first season, a total of *n* = 177,811 patients were analyzed, of whom n = 134,633 (75.7%) were classified at risk due to age ≥ 65 years, and *n* = 85,938 (48.3%) due to chronic disease. The overall VUR ranged between 21.8% (2018/2019 season) and 29.1% (2020/2021 season). For all groups, except for patients living in nursing homes, the highest VUR was observed during the first pandemic season (Table 2A).

**Table 1:** Baseline characteristics

Influenza Season		2018/2019	2019/2020	2020/2021	2021/2022
n		177,811	179,359	184,672	190,890
Age mean (SD)		67.75 (12.28)	68.03 (12.13)	68.06 (12.14)	67.94 (12.30)
Sex (%)	Female	89,295 (50.2)^c^	90,652 (50.5) ^d^	93,791 (50.8)^c^	97,515 (51.1)^d^
Swiss Nationality (%)		129,572 (72.9)	130,542 (72.8)	134,077 (72.6)	138,037 (72.3)
Language in residence area (%)	Abroad	1052 (0.6)	1166 (0.7)	1267 (0.7)	1453 (0.8)
	French	104,506 (58.8)	104,643 (58.3)	106,711 (57.8)	109,238 (57.2)
	German	65,121 (36.6)	66,422 (37.0)	69,396 (37.6)	72,619 (38.0)
	Italian	7132 (4.0)	7128 (4.0)	7298 (4.0)	7580 (4.0)
Deductible level^a^ (%)	Level 1	147,624 (83.0)	148,425 (82.8)	151,527 (82.1)	155,882 (81.7)
	Level 2	16,872 (9.5)	16,828 (9.4)	17,621 (9.5)	17,941 (9.4)
	Level 3	13,315 (7.5)	14,106 (7.9)	15,524 (8.4)	17,067 (8.9)
Insurance model (%)	Free-choice	82,842 (46.6)	80,942 (45.1)	80,076 (43.4)	79,456 (41.6)
	Network	17,234 (9.7)	17,467 (9.7)	18,344 (9.9)	19,205 (10.1)
	Family Medicine practice	51,677 (29.1)	53,079 (29.6)	55,050 (29.8)	57,509 (30.1)
	Telemedicine	26,058 (14.7)	27,871 (15.5)	31,202 (16.9)	34,720 (18.2)
Number of GP consultations, median [IQR]		3 [0,6]	3 [0,6]	3 [0,6]	2 [0,5]
Number of specialist consultations, median [IQR]		3 [0,7]	2 [0,6]	3 [1, 7]	3 [1, 7]
Risk groups (%)	Age ≥ 65 independent of comorbidity	134,633 (75.7)	137,138 (76.5)	140,934 (76.3)	144,807 (75.9)
	At least one comorbidity^b^ (%)	85,938 (48.3)	85,581 (47.7)	88,793 (48.1)	92,580 (48.5)
	Age ≥ 65, comorbidity	42,760 (24.0)	43,360 (24.2)	45,055 ( 24.4)	46,497 ( 24.4)
	Age ≥ 65, no comorbidity	91,873 (51.7)	93,778 (52.3)	95,879 ( 51.9)	98,310 ( 51.5)
	Age < 65, comorbidity	43,178 (24.3)	42,221 (23.5)	43,738 ( 23.7)	46,083 ( 24.1)
Living in nursing home (%)		8457 (4.8)	8622 (4.8)	8821 (4.8)	8259 (4.3)

^a^ Deductible level ≤ 500 Swiss Francs (level 1), 501–1500 CHF (level 2), 1501–2500 CHF (level 3), 1 Swiss Franc = 1.0824 USD (as of 01/01/2023). 2: Comorbidities of interest are: chronic heart-, liver-, lung-, and kidney-diseases; diabetes mellitus; cerebral diseases; patients with cancer, lymphoma, leukemia or myeloma, and immunocompromised patients with autoimmune diseases, drug immunosuppression, human immunodeficiency viruses positive, or transplantations

^b^ At least one chronic disease from: heart, liver, lung, cerebral and kidney diseases, diabetes mellitus or immunocompromised patients with autoimmune diseases, drug immunosuppression, human immunodeficiency viruses (HIV), cancer, lymphoma, leukemia or myeloma or transplantations

^c^ missing: n = 10

^d^ missing: n = 11

*SD* Standard deviation, *IQR* Interquartile Range, *GP* General practitioners

**Table 2:** Influenza vaccination uptake (VUR) rates

Influenza season	2018/2019	2019/2020	Change (%)	2020/2021	Change (%)	2021/2022	Change (%)
A: All patients (cross sectional)							
Overall	21.8	23.4	7.3%	29.1	24.4%	25.2	− 13.4%
Patients with chronic diseases	26.3	28.5	8.4%	33.7	18.2%	29.2	− 13.3%
Patients ≥ 65	23.6	25.2	6.8%	31.4	24.6%	27.9	− 11.1%
Patients living in nursing homes	46.4	46.9	1.1%	48.4	3.2%	48.9	1.0%
B: Cohort subgroup							
Overall	21.9	25.1	14.6%	33.0	31.5%	30.2	− 8.5%
Patients with chronic diseases	28.3	31.9	12.7%	39.4	23.5%	35.7	-9.4%
Patients ≥ 65	22.6	25.9	14.6%	34.3	32.4%	31.7	− 7.6%
Patients living in nursing homes	43.8	47.0	7.3%	50.8	8.1%	50.0	− 1.6%

Results were presented as percentage (%)

Change was defined as the rate of change of Influenza uptakes from a season to the following one i.e. (uptake 2019/2020-uptake 2018/2019) / uptake 2018/2019

Cohort subgroup.

Data from *n* = 138,526 patients were available throughout all four seasons. The cohort had different characteristics compared to the non-cohort patients. Specifically, a higher percentage of patients were aged ≥ 65 years, fewer patients were male, and the cohort showed lower deductibles. Additionally, patients in the cohort more frequently had an insurance model that allowed free choice of physicians and showed more health care provider visits (Supplemental Table 3 and 4). Overall VUR rates in the cohort ranged from 21.9% (2018/2019) to 33.0% (2020/2021) (Table 2B).

**Fig. 1:**
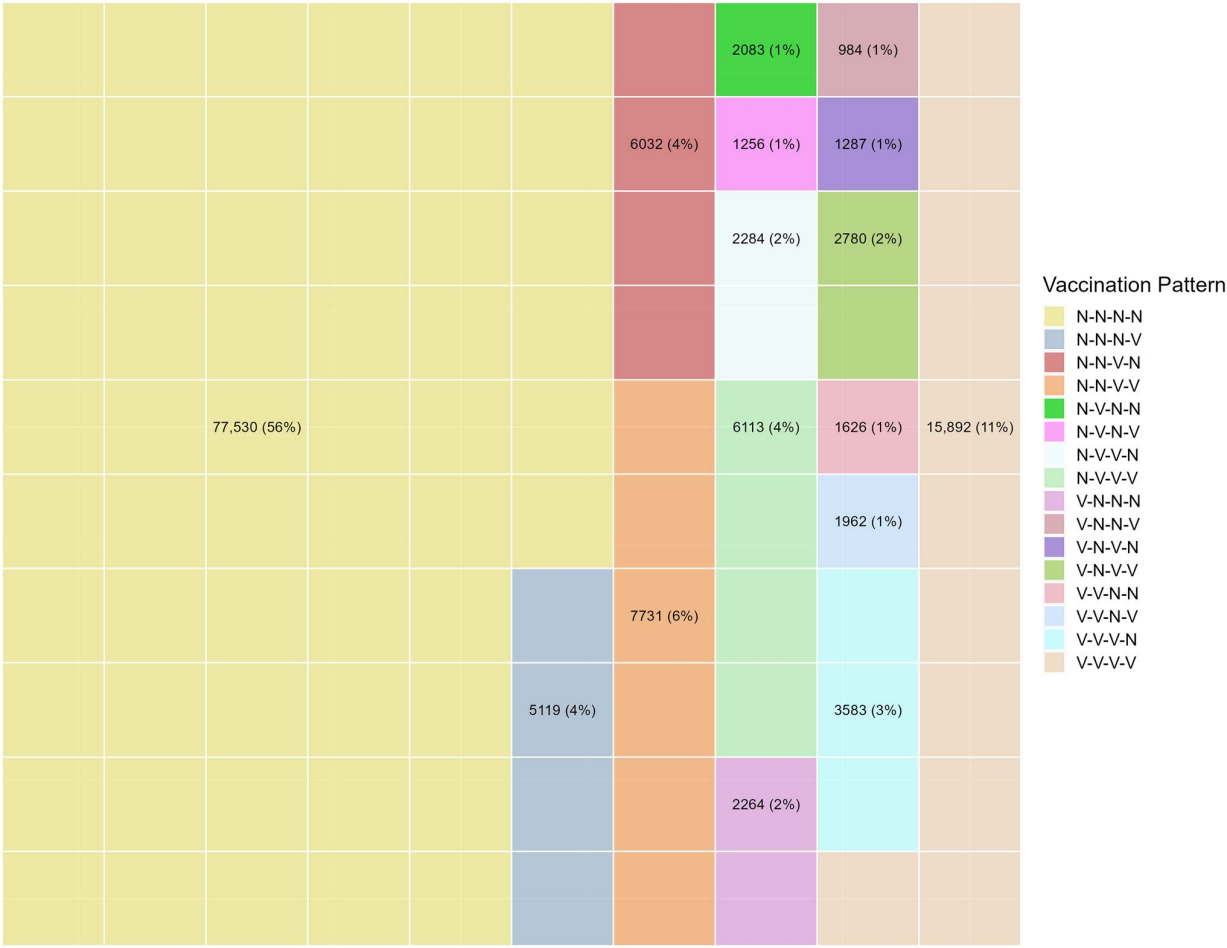
Vaccination behavior in the cohort subgroup across all four influenza seasons (2018/2019—2021/2022)

Different individual vaccination patterns during the observation period are presented in Fig. 1. Only *n* = 15,892 (11%) patients were vaccinated in all observed seasons. A subgroup of 13,763 (14.3% of previously unvaccinated patients), were vaccinated for the first time in the first pandemic season and *n* = 7731 (56.1%) of these were vaccinated in the second pandemic season too. Compared to the rest of the cohort, FTVP were more common ≥ 65 years of age (85.0% vs. 82.9%, p < 0.001), had more GP and specialists consultations (median 4 vs. 3, p < 0.001) and had more often a comorbidity (48.6% vs 45.9%, p < 0.001) (Supplemental Table 5).

Multivariable logistic regression analysis identified factors associated with vaccination status in the second pandemic season (Table 3). The strongest predictor for vaccination was history of vaccination in the previous seasons (OR 56.20 95%CI 53.62–58.90, p < 0.001 for being vaccinated in all three previous seasons). In contrast, being Swiss citizen (OR: 0.95, 95%CI 0.92–0.99, p = 0.006) or having a chronic heart disease (OR: 0.91, 95%CI 0.87–0.97, p = 0.001) was associated with lower odds of being vaccinated in the second pandemic season. The latter was associated in the opposite direction in univariable model. For each predictor in multivariable analysis, the VIF was not greater than 1.25, excluding the presence of bias due to multicollinearity.

**Table 3:** Logistic regression analysis. Outcome: being vaccinated during season 2021/2022. Cohort subgroup patients, n = 138,526

Factor	Not vaccinated	Vaccinated	Univariable	Multivariable
	N = 96,689	N = 41,837	OR (95% CI, p-value)	OR (95% CI, p-value)
Sex				
Female	49,606 (51.3)	21,431 (51.2)	–	–
Male	47,077 (48.7)	20,403 (48.8)	1.00 (0.98–1.03, p = 0.787)	1.04 (1.01–1.07, p = 0.013)
Age	70.2 (10.9)^a^	74.3 (10.0)^a^	1.04 (1.04–1.04, p < 0.001)	1.02 (1.02–1.02, p < 0.001)
Living in nursing home				
No	92,819 (96.0)	37,961 (90.7)	–	–
Yes	3870 (4.0)	3876 (9.3)	2.45 (2.34–2.56, p < 0.001)	1.12 (1.05–1.19, p = 0.001)
History of previous vaccination				
None (never vaccinated)	77,530 (80.2)	5119 (12.2)	–	–
One vaccinations	10,379 (10.7)	9971 (23.8)	14.54 (13.98–15.12, p < 0.001)	13.46 (12.93–14.01, p < 0.001)
Two vaccinations	5197 (5.4)	10,855 (25.9)	31.61 (30.26–33.01, p < 0.001)	27.78 (26.57–29.04, p < 0.001)
Three vaccinations (always)	3583 (3.7)	15,892 (38.0)	67.09 (64.07–70.24, p < 0.001)	56.20 (53.62–58.90, p < 0.001)
Immunocompromised patient^b^				
No	83,025 (85.9)	34,693 (82.9)	–	–
Yes	13,664 (14.1)	7144 (17.1)	1.25 (1.21–1.29, p < 0.001)	1.02 (0.98–1.07, p = 0.333)
Lung disease				
No	90,292 (93.4)	37,157 (88.8)	–	–
Yes	6397 (6.6)	4680 (11.2)	1.78 (1.71–1.85, p < 0.001)	1.23 (1.16–1.30, p < 0.001)
Heart disease				
No	90,086 (93.2)	37,112 (88.7)	–	-
Yes	6603 (6.8)	4725 (11.3)	1.74 (1.67–1.81, p < 0.001)	0.91 (0.87–0.97, p = 0.001)
Diabetes mellitus				
No	78,926 (81.6)	32,846 (78.5)	–	–
Yes	17,763 (18.4)	8991 (21.5)	1.22 (1.18–1.25, p < 0.001)	1.13 (1.09–1.18, p < 0.001)
Swiss nationality				
No	23,975 (24.8)	10,982 (26.2)	–	–
Yes	72,714 (75.2)	30,855 (73.8)	0.93 (0.90–0.95, p < 0.001)	0.95 (0.92–0.99, p = 0.006)
Number of GP consultations	2 [0,4]^c^	4 [2, 7] c	1.11 (1.10–1.11, p < 0.001)	1.06 (1.06–1.07, p < 0.001)
Number of specialist consultations	2 [0,6]^c^	4 [1, 8] c	1.03 (1.03–1.04, p < 0.001)	1.02 (1.02–1.02, p < 0.001)

Patient variables are reported as absolute numbers (percentage) if not stated else

^a^ mean (standard deviation) reported

^b^ Patients with autoimmune diseases, drug immunosuppression, human immunodeficiency viruses (HIV), cancer, lymphoma, leukemia or myeloma or transplantations

^c^ median (interquartile range) reported

*CI* Confidence interval, *GP* General Practice, *OR* Odds ratio

The waffle chart shows the vaccination patterns over the four seasons analyzed in the cohort subgroup. Each square represents 1% of patients in the cohort subgroup. Given the dichotomous outcome of vaccination, there are 16 possible vaccination patterns. All 16 possible combinations of vaccinated (V)/unvaccinated (N) across the four observed influenza seasons (2018/2019—2021/2022) are shown. For example, N–N-V–N indicates that *n* = 6032 (4%) patients were vaccinated only in the third (2020/2021) influenza season.

## Discussion

In this retrospective study, we investigated influenza VUR in Switzerland for at-risk patients before and during the Covid-19 pandemic. The highest VUR occurred during the first pandemic season. Prior vaccination (one, two, or three vaccinations) was the most significant predictor for vaccination in the following season. Only a minority of at-risk patients, who were not vaccinated during the pre-pandemic seasons, received the vaccine during the first pandemic season. These patients were predominantly older, had more chronic diseases, and had more frequent visits to their healthcare professionals.

### Overall influenza vaccination uptake rates

In all four analyzed influenza seasons, VUR among at-risk patients was below the World Health Organization target of 75% [22]. We found increasing VUR with the highest VUR in the 2020/2021 season. In this season, Covid-19 vaccination was not yet available for the majority of the population although older patients and those with pre-existing conditions had been prioritized for vaccination [23]. With exception of patients living in nursing homes, who already showed the highest VUR, there was a relative increase in VUR from the 2019/2020 pandemic-start season to the following pandemic season by 24% overall. Although some countries reported declining VUR in the 2020/2021 season [24], increasing VUR in the 2020/2021 season was a common phenomenon [25, 26]. This could be explained by a different perception of the threat of infectious diseases and by increased public health efforts to raise awareness in the general population, especially at-risk patients [13, 27, 28].

In the years before the pandemic, VUR declined in Switzerland [11] and we found a decrease in VUR again from the 2020/2021 to the 2021/2022 season. The Covid-19 wave in the 2021/2022 season differed in many aspects from the previous season. Although Covid-19 case numbers were higher in the second pandemic season, mortality rates were lower [29]. Patients willing to be vaccinated against Covid-19 were vaccinated and the phenomenon of pandemic fatigue arises [30]. In addition, driven by the hygiene measures and behavior change during the pandemic, the influenza season 2020/2021 was relatively weak [15, 31, 32]. These circumstances may have led to a lower individual perception of risk and the subsequent decision to avoid the influenza vaccination.

### Cohort subgroup

We determined vaccination uptake at patient level over four influenza seasons. We found that the majority of patients were never vaccinated and only 11% were vaccinated every season. A similar low rate of always vaccinated was recently reported in patients aged ≥ 65 years of age from the United States [14]. Approximately 40% of patients show alternating vaccination pattern and patients generally report a variety of reasons for being vaccinated or not vaccinated [33]. This pattern reflects the overall low VUR in Switzerland [10, 11]. Possible reasons such as mistrust, fear of side effects, or a perceived lack of benefit, as reported in other studies [27, 34], might contribute to explain this low proportion in Switzerland but it remains unclear.

Of great concern is that only a minority of previously eligible but unvaccinated patients were vaccinated in the 2020/2021 season. The proportion of FTVP is lower compared to similar studies from Canada or the UK [13, 27]. FTVP were more likely to be aged ≥ 65 years, to have chronic diseases and to have more health care provider visits compared to the rest of the cohort. These variables were identified as predictors for vaccination in our analysis. In addition, health authorities specifically addressed these patient groups during the first pandemic year in 2020. Nevertheless, we could show that more than half of the patients who were vaccinated for the first time in the 2020/2021 season also received the vaccination in the 2021/2022 season. As confirmed by our analysis, vaccination history has been identified as a key predictor for future vaccination [12–14]. These findings underline the importance of knowing the vaccination status in previous years to encourage patients to re-vaccinate. In the upcoming influenza seasons, health care providers can take advantage of the temporary increased acceptance of influenza vaccination after the Covid-19 pandemic to foster VUR.

National surveillance reports VUR based on representative telephone surveys. Reported VUR in people aged ≥ 65 years was 28%, 38% and 37% for the seasons from 2019/20 to 2021/2022, respectively. VUR in people with chronic diseases was reported to be 27%, 37%, and 35% respectively [35]. The VUR observed in this study was lower than national surveillance data. We attribute this discrepancy primarily to methodological factors. The difference was less pronounced in the cohort subgroup and, in particular, patients aged ≥ 65 years had similar VUR. Therefore, the patients included in the cohort differ from the other patients. In fact, we found that several factors identified as predictors of vaccination were more prevalent in the cohort. Moreover, the two groups might differ in other unmeasured factors that influenced vaccination behavior. Additional research is required to provide a more comprehensive description and explanation of this phenomenon. Regardless of the population studied, the calculated VUR confirmed the importance of ongoing national efforts to increase vaccination uptake. Our VUR in at-risk patients, as well as the associations identified in the regression analysis, can support the FOPH and other stakeholders in future interventions to improve national VUR.

In contrast to immunocompromised patients or patients with chronic lung disease, we found that patients with chronic heart disease were less likely to be vaccinated, after adjusting for vaccination history, age and number of consultations at the same time. Though this could be due to a problem of variable selection, it seems that multicollinearity was not present in our model, but there was just a moderate pairwise correlation between some predictors. Our case is an example of paradoxes whose explanation and solution lie more in causal reasoning rather than statistical criteria [36] and this would require further investigation. Although having one of the other chronic diseases was associated with increased odds of being vaccinated, our calculated odds were barely above one. This finding is similar to the results of a recent study which showed that patients with chronic diseases are not more willing to be vaccinated against Covid-19 [37].

### Strengths and limitations

Our study has limitations. First, we could not analyze the effect of the individual Covid-19 vaccination status, since the federal government covered directly vaccination costs during the pandemic without involving health insurance companies. Therefore, this information was not available to GM. Second, VUR in our analysis were below the VUR reported by the national surveillance or recent studies [33, 35]. Our criteria to identify patients with chronic diseases were chosen to detect as many patients as possible. We cannot exclude the possibility that individual patients with chronic diseases were not identified as being at-risk in routine clinical practice. In addition, vaccinations at the workplace or in pharmacies are often not reported to insurance companies. In the 2017/2018 season, only 1.4% of all influenza vaccine doses were administered in pharmacies [38]. We did not have any data for later seasons so we could not estimate the proportion of vaccinations that were not reported to the insurance company. However, the large at-risk population analyzed in this study and the risk that the insurance company is unaware of the true vaccination status may have led to an underestimation of true VUR. In contrast, self-reported vaccination status, as used in national surveillance, is known to overestimate true vaccination status to some extent [39, 40]. Third, due to the observational and not experimental design of our study, we could not investigate causality and therefore explain our change in association result for heart-disease patients in multivariable model.

## Conclusion

Our analysis confirmed an overall low influenza VUR and that only a minority of previously eligible but unvaccinated at-risk patients were vaccinated for the first time in the first pandemic season. As previous vaccinations are the most important predictor for subsequent vaccinations, health care professionals should actively address at-risk patients’ vaccination history in order to recommend vaccination.

### Supplementary Information

Below is the link to the electronic supplementary material.

Supplementary file1 (DOCX 51 KB)

## Data Availability

Research data are not shared.
