# Supplementary material for: Influenza vaccination patterns among at-risk patients during the Covid-19 pandemic—a retrospective cross-sectional study based on claims data
Source: Infection. 2024 Feb 1;52(4):1287–95. doi: 10.1007/s15010-024-02175-3 (PMC11289170; doi:10.1007/s15010-024-02175-3)
Supplement: Supplementary file 1 — Supplementary file1 (DOCX 51 KB) [file 15010_2024_2175_MOESM1_ESM.docx]

Supplemental file

Title: Influenza vaccination patterns among at-risk patients during the Covid-19 pandemic – a retrospective cross-sectional study based on claims data.

Journal: Infection

| Supplemental table 1: Identification of chronic diseases | | |
| --- | --- | --- |
| **Chronic disease** | **Code** | |
| By medication using Pharmaceutical Cost Group, PCG code^1^ or ATC code^2^ | | |
| Chronic heart disease | | CAR, HYP |
| Chronic lung disease | | AST, COP, ZFP |
| Chronic liver disease | |  |
| Chronic kidney disease | | NIE |
| Cerebral disease | | MSK, ZNS |
| Diabetes mellitus | | DM1, DM2, DM2+ |
| Lymphoma, leukemia, myeloma | |  |
| Transplantations | | TRA |
| Autoimmune disease or drug immunosuppression | | AIK, RHE. ATC: L01, L04 |
| HIV | | HIV |
| Cancer | | KRE, KRK |
| By in hospital stay diagnosis, Diagnosis-related group, DRG^3^ | | |
| Chronic heart disease | | F01A, F01B, F01C, F01D, F01E, F01F, F02A, F02B, F03A, F03B, F03C, F03D, F03E, F06A, F06B, F06C, F06D, F12A, F12B, F12C, F12D, F12E, F12F, F17A, F17B, F18A, F18B, F24A, F24B, F24C, F24D, F24E, F24F, F60A, F60B, F62A, F62B, F62C, F62D, F66A, F66B, F67A, F67B, F68A, F68B, F69A, F69B, F98A, F98B, F98C |
| Chronic lung disease | | E60A, E60B, E65A, E65B, E65C, E65D, E65E |
| Chronic liver disease | | H09A, H09B, H09C, H60A, H60B, H60C |
| Chronic kidney disease | | L60A, L60B, L60C, L60D, L60E |
| Cerebral disease | | B67A, B67B, B68A, B68B, B68C, B69A, B69B, B69C, B69D, B70A, B70B, B70C, B70D, B70E, B70F, B70G, B70J, B70K |
| Diabetes mellitus | |  |
| Lymphoma, leukemia, myeloma | | R60A, R60B, R60C, R60D, R61A, R61B, R61C, R61D, R63A, R63B, R63C, R63D, R63E |
| Transplantations | | A01A, A01B, A02Z, A04A, A04B, A05A, A05B, A60A, A60B, A60C |
| Autoimmune disease or drug immunosuppression | |  |
| HIV | | S01Z, S62Z, S63A, S63B, S65Z |
| Cancer | | A93A, A93B, A93C, A93D, B16Z, D35Z, D60A, D60B, E03Z, E05B, E08A, E08B, E71A, E71B, G16Z, G17A, G19A, G29B, G60A, G60B, H61A, H61B, I54A, I54B, I65A, I65B, J01A, J01B, J06A, J06B, J18A, J18B, J23A, J23B, J62A, J62B, K15A, K15B, L12Z, L62A, L62B, M09A, M09B, M10Z, M60A, M60B, N01A, N01B, N01C, N16Z, N60A, N60B, R01A, R01B, R01C, R01D, R01E, R50A, R50B, R50C, R62A, R62B, R62C, R65A, R65B, R65C |
| By medical services, Procedure codes, TARMED^4^ | | |
| Chronic heart disease | | 17.1110, 17.1210, 17.1510, 17.1520, 17.1540, 17.1560, 17.1570, 17.1590, 17.1600, 17.1610, 17.1620, 17.1630, 17.1640, 17.1650, 17.1660, 18.0110, 18.0160, 18.0170, 18.0210, 18.0230, 18.0240, 18.0250, 18.0260, 18.0300, 18.0310, 18.0330, 18.0340 |
| Chronic lung disease | |  |
| Chronic liver disease | | 19.0210 + (19.0260 or 19.0270 or 19.0280 or 19.0290), 19.0410 + (19.0460 or 19.0470 or 19.0480 or 19.0490) |
| Chronic kidney disease | |  |
| Cerebral disease | |  |
| Diabetes mellitus | |  |
| Lymphoma, leukemia, myeloma | |  |
| Transplantations | | 21.0960 |
| Autoimmune disease or drug immunosuppression | |  |
| HIV | |  |
| Cancer | | 04.0320, 04.0330, 04.0340, 04.0350, 05.1220, 05.1240, 05.1250, 05.1260, 05.1270, 05.1280, 05.1290, 05.1300, 05.1310, 05.1320, 05.1330, 05.1340, 05.1350, 05.1360, 05.1370, 05.1380, 05.1390, 05.1400, 05.1410, 05.1420, 05.1430, 05.1440, 07.1240, 07.1260, 10.1230, 10.1240, 11.0270, 11.0300, 11.0310, 11.0320, 11.0800, 11.0810,  12.0330,  12.0360,  12.0370,  12.0390,  14.0360 + 14.0370, 16.0150, 16.0250, 16.0840 + 16.0940, 21.0510 + 21.0600, 21.0920, 21.0970, 22.0360 + 22.0470, 23.0140, 23.0150, 23.0190, 23.0200, 23.0210, 24.0560, 24.0570, 24.1420, 24.1440, 24.1820, 24.2270, 24.2280, 24.2640, 24.4130, 24.8570, 26.0030, 26.0050, 26.0230, 26.0340, 26.0350, 26.0420, 26.0450, 32. |
| 1: Health insurance: Risk compensation. <https://www.bag.admin.ch/bag/en/home/versicherungen/krankenversicherung/krankenversicherung-versicherer-aufsicht/risikoausgleich.html>,  2: WHO ATC Codes: https://www.whocc.no/atc_ddd_index/  3: SwissDRG AG. <https://www.swissdrg.org/de>  4: Outpatients Tarifs. [https://www.fmh.ch/themen/ambulante-tarife.cfm#](https://www.fmh.ch/themen/ambulante-tarife.cfm) | | |

| Supplemental table 2 – Proportion of chronic diseases. Cross sectional dataset | | | | |
| --- | --- | --- | --- | --- |
| Influenza season | 2018/2019 | 2019/2020 | 2020/2021 | 2021/2022 |
| N of patients | 177,811 | 179,359 | 184,672 | 190,890 |
| Number of all comorbidities, mean SD* | 1.40 (1.27) | 1.42 (1.29) | 1.44 (1.29) | 1.44 (1.31) |
| Lung diseases | 14,929 (8.4) | 14,934 (8.3) | 16,021 (8.7) | 15,752 (8.2) |
| Cardiovascular diseases | 14,150 (7.9) | 13,781 (7.7) | 14,039 (7.6) | 13,704 (7.2) |
| Diabetes | 32,198 (18.1) | 32,096 (17.9) | 32,987 (17.8) | 34,393 (18.0) |
| Drug immunosuppression & autoimmune disease | 20,560 (11.6) | 20,955 (11.7) | 21,973 (11.9) | 25,641 (13.4) |
| Transplantations | 872 (0.5) | 864 (0.5) | 851 (0.5) | 826 (0.4) |
| Cancer | 8403 (4.7) | 8306 (4.6) | 9091 (4.9) | 9133 (4.8) |
| HIV** | 2072 (1.2) | 2075 (1.2) | 2111 (1.1) | 2073 (1.1) |
| Cerebral diseases | 4077 (2.3) | 4100 (2.3) | 4031 (2.2) | 3941 (2.1) |
| Renal insufficiency | 600 (0.3) | 632 (0.4) | 683 (0.4) | 691 (0.4) |
| Liver diseases | 361 (0.2) | 327 (0.2) | 347 (0.2) | 325 (0.2) |
| Lymphoma, leukaemia or myeloma | 344 (0.2) | 315 (0.2) | 328 (0.2) | 304 (0.2) |
| All numbers are reported as absolute numbers and percentage if not stated otherwise  *: Based on positive Pharmaceutical Cost Group (PCG). Range in analysed patients: 0-9  **: Human immunodeficiency viruses | | | | |

| Supplemental table 3: Baseline characteristics – cohort subgroup | | | | | |
| --- | --- | --- | --- | --- | --- |
| Influenza season |  | 2018/2019 | 2019/2020 | 2020/2021 | 2021/2022 |
| Number of patients |  | 138,526 | | | |
| Age | ≥ 65 years | 111,732 (80.7) | 113,629 (82.0) | 115,119 (83.1) | 116,461 ( 84.1) |
| Sex | Female | 71,037 (51.3) missing=9 | | | |
| Living in nursing home | Yes | 3741 (2.7) | 4832 (3.5) | 6845 (4.9) | 7746 (5.6) |
| Swiss citizenship |  | 103,039 (74.4) | 103,238 (74.5) | 103,426 (74.7) | 103,569 (74.8) |
| Language in residence area | abroad | 812 (0.6) | 877 (0.6) | 931 (0.7) | 1012 (0.7) |
|  | French | 82,232 (59.4) | 82,171 (59.3) | 82,118 (59.3) | 82,073 (59.2) |
|  | German | 50,008 (36.1) | 50,009 (36.1) | 50,003 (36.1) | 49,963 (36.1) |
|  | Italian | 5474 (4.0) | 5469 (3.9) | 5474 (4.0) | 5478 (4.0) |
| Deductible level* | Level 1 | 115,478 (83.4) | 116,424 (84.0) | 116,903 (84.4) | 117,825 (85.1) |
|  | Level 2 | 13,238 ( 9.6) | 12,615 (9.1) | 12,329 (8.9) | 11,784 (8.5) |
|  | Level 3 | 9810 (7.1) | 9487 (6.8) | 9294 (6.7) | 8917 (6.4) |
| Insurance model | Free-choice | 65,339 (47.2) | 64,095 (46.3) | 62,962 (45.5) | 62,334 (45.0) |
|  | Network | 13,258 (9.6) | 13,201 (9.5) | 13,230 (9.6) | 13,322 (9.6) |
|  | Family medicine practice | 39,603 (28.6) | 40,612 (29.3) | 41,112 (29.7) | 41,383 (29.9) |
|  | Telemedicine | 20,326 (14.7) | 20,618 (14.9) | 21,222 (15.3) | 21,487 (15.5) |
| Number of GP consultations, median [IQR] |  | 3 [1,6] | 3 [0,6] | 3 [0,6] | 2 [0,5] |
| Number of specialist consultations, median [IQR] |  | 3 [1,7] | 3 [1,7] | 3 [1,7] | 3 [1,7] |
| Number of all comorbidities**, mean (SD) |  | 1.37 (1.27) | 1.46 (1.29) | 1.52 (1.32) | 1.59 (1.34) |
| Lung diseases |  | 9573 (6.9) | 10,118 (7.3) | 10,687 (7.7) | 11,077 (8.0) |
| Cardiovascular diseases |  | 9384 (6.8) | 9954 (7.2) | 10,925 (7.9) | 11,328 (8.2) |
| Diabetes |  | 25,069 (18.1) | 25,641 (18.5) | 26,144 (18.9) | 26,754 (19.3) |
| Drug immunosuppression & autoimmune disease |  | 13,636 (9.8) | 14,211 (10.3) | 15,158 (10.9) | 16,371 (11.8) |
| Transplantations |  | 709 (0.5) | 749 (0.5) | 748 (0.5) | 728 (0.5) |
| Cancer |  | 3891 (2.8) | 4315 (3.1) | 5330 (3.8) | 6198 (4.5) |
| HIV*** |  | 1709 (1.2) | 1717 (1.2) | 1706 (1.2) | 1683 (1.2) |
| Cerebral diseases |  | 2532 (1.8) | 2645 (1.9) | 2723 (2.0) | 2705 (2.0) |
| Renal insufficiency |  | 329 (0.2) | 365 (0.3) | 455 (0.3) | 553 (0.4) |
| Liver diseases |  | 128 (0.1) | 115 (0.1) | 136 (0.1) | 166 (0.1) |
| Lymphoma, leukaemia or myeloma |  | 139 (0.1) | 112 (0.1) | 165 (0.1) | 216 (0.2) |
| All numbers are reported as absolute numbers and percentage if not stated otherwise. IQR: Interquartile Range; SD: Standard Deviation;  *: level 1: ≤ 500 Swiss francs (CHF); level 2: 501 – 1500 CHF: level 3: 1501 – 2500 CHF  **: Based on positive Pharmaceutical Cost Group (PCG). Range in analysed patients: 0-4  ***: Human immunodeficiency viruses | | | | | |

| Supplemental table 4: Comparison of baseline characteristics between the cohort subgroup and cross-sectional patients (CS) | | | | | | | | | | | | |
| --- | --- | --- | --- | --- | --- | --- | --- | --- | --- | --- | --- | --- |
| Influenza Season | 2018/ 2019 | | | 2019/2020 | | | 2020/2021 | | | 2021/2022 | | |
| Subgroups | CS | Cohort | p | CS | Cohort | p | CS | Cohort | p | CS | Cohort | p |
| n | 39,285 | 138,526 |  | 40,833 | 138,526 |  | 46,146 | 138,526 |  | 52,364 | 138,526 |  |
| Age, ≥ 65 years | 22,901 (58.3) | 111,732 (80.7) | <0.001 | 23,509  (57.6) | 113,629  (82.0) | <0.001 | 25,815 (55.9) | 115,119 (83.1) | <0.001 | 28,346 (54.1) | 116,461 (84.1) | <0.001 |
| Swiss citizenship | 26,533  (67.5) | 103,039  (74.4) | <0.001 | 27,304 (66.9) | 103,238 (74.5) | <0.001 | 30,651 (66.4) | 103,426 (74.7) | <0.001 | 34,468 (65.8) | 103,569 (74.8) | <0.001 |
| Vaccinated | 8417  (21.4) | 30,378  (21.9) | 0.033 | 7240  (17.7) | 34813  (25.1) | <0.001 | 7958  (17.2) | 45,702  (33.0) | <0.001 | 6343  (12.1) | 41,837  (30.2) | <0.001 |
| Language in residence area |  |  | <0.001 |  |  | <0.001 |  |  | <0.001 |  |  | <0.001 |
| Abroad | 240  (0.6) | 812  (0.6) |  | 289  (0.7) | 877  (0.6) |  | 336  (0.7) | 931  (0.7) |  | 441  (0.8) | 1012  (0.7) |  |
| French | 22,274  (56.7) | 82,232  (59.4) |  | 22,472  (55.0) | 82,171  (59.3) |  | 24,593 (53.3) | 82,118 (59.3) |  | 27,165 (51.9) | 82,073  (59.2) |  |
| German | 15,113  (38.5) | 50,008  (36.1) |  | 16,413  (40.2) | 50,009  (36.1) |  | 19,393 (42.0) | 50,003 (36.1) |  | 22,656 (43.3) | 49,963  (36.1) |  |
| Italian | 1658  (4.2) | 5474  (4.0) |  | 1659  (4.1) | 5469  (3.9) |  | 1824  (4.0) | 5474  (4.0) |  | 2102  (4.0) | 5478  (4.0) |  |
| Male sex | 21,026  (53.5) | 67,480  (48.7) | <0.001 | 21,216 (52.0) | 67,480 (48.7) | <0.001 | 23,391 (50.7) | 67,480 (48.7) | <0.001 | 25,884 (49.4) | 67,480 (48.7) | 0.005 |
| At least one comorbidity | 26,636  (67.8) | 59,302  (42.8) | <0.001 | 24,455 (59.9) | 61,126 (44.1) | <0.001 | 24,834 (53.8) | 63,959 (46.2) | <0.001 | 26,228 (50.1) | 66,352 (47.9) | <0.001 |
| Risk group |  |  | <0.001 |  |  | <0.001 |  |  | <0.001 |  |  | <0.001 |
| comorbidity + age | 10,252  (26.1) | 32,508  (23.5) |  | 7131  (17.5) | 36,229  (26.2) |  | 4517  (9.8) | 40696  (29.4) |  | 2210  (4.2) | 44,287  (32.0) |  |
| only age | 12,649  (32.2) | 79,224  (57.2) |  | 16,378 (40.1) | 77,400 (55.9) |  | 21281  (46.0) | 74430  (53.7) |  | 26,136 (49.9) | 72,174 (52.1) |  |
| only comorbidity | 16,384  (41.7) | 26,794  (19.3) |  | 17324 (42.4) | 24,897 (18.0) |  | 20470  (44.2) | 23480  (16.9) |  | 24,018 (45.9) | 22065 ( 15.9) |  |
| Deductible level* |  |  | <0.001 |  |  | <0.001 |  |  | <0.001 |  |  | <0.001 |
| Level 1 | 32,146  (81.8) | 115,478 (83.4) |  | 32,001 (78.4) | 116,424 (84.0) |  | 34,624 (75.0) | 116,903 (84.4) |  | 38,057 (72.7) | 117,825 (85.1) |  |
| Level 2 | 3634  (9.3) | 13,238  (9.6) |  | 4213  (10.3) | 12,615  (9.1) |  | 5292  (11.5) | 12,329  (8.9) |  | 6157  (11.8) | 11,784 (8.5) |  |
| Level 3 | 3505  (8.9) | 9810  (7.1) |  | 4619  (11.3) | 9487  (6.8) |  | 6230  (13.5) | 9294  (6.7) |  | 8150  (15.6) | 8917  (6.4) |  |
| Insurance model |  |  | <0.001 |  |  | <0.001 |  |  | <0.001 |  |  | <0.001 |
| Free choice | 17,503  (44.6) | 65,339 (47.2) |  | 16,847 (41.3) | 64,095 (46.3) |  | 17,114 (37.1) | 62,962  (45.5) |  | 17,122 (32.7) | 62,334 (45.0) |  |
| Network | 3976  (10.1) | 13,258 (9.6) |  | 4266  (10.4) | 13,201  (9.5) |  | 5114  (11.1) | 13,230  (9.6) |  | 5883  (11.2) | 13,322 (9.6) |  |
| Family medicine | 12,074  (30.7) | 39,603 (28.6) |  | 12,467 (30.5) | 40,612 (29.3) |  | 13,938 (30.2) | 41,112 (29.7) |  | 16,126 (30.8) | 41,383  (29.9) |  |
| Telemed | 5732  (14.6) | 20,326 (14.7) |  | 7253  (17.8) | 20,618 (14.9) |  | 9980  (21.6) | 21,222  (15.3) |  | 13,233 (25.3) | 21,487 (15.5) |  |
| Number of all comorbidities**, mean (SD) | 1.49  (1.28) | 1.37  (1.27) | <0.001 | 1.30  (1.26) | 1.46  (1.29) | <0.001 | 1.17  (1.19) | 1.52  (1.32) | <0.001 | 1.05  (1.13) | 1.59  (1.34) | <0.001 |
| Number of GP consultations, median [IQR] | 2.00  [0.00, 6.00] | 3.00  [1.00, 6.00] | <0.001 | 2.00  [0.00, 5.00] | 3.00  [0.00, 6.00] | <0.001 | 2.00  [0.00, 5.00] | 3.00  [0.00, 6.00] | <0.001 | 1.00  [0.00, 4.00] | 2.00  [0.00, 5.00] | <0.001 |
| Number of specialist consultations, median [IQR] | 2.00  [0.00, 6.00] | 3.00  [1.00, 7.00] | <0.001 | 2.00  [0.00, 6.00] | 3.00  [1.00, 7.00] | <0.001 | 2.00  [0.00, 6.00] | 3.00  [1.00, 7.00] | <0.001 | 2.00  [0.00, 6.00] | 3.00  [1.00, 7.00] | <0.001 |
| Living in nursing home | 4716  (12.0) | 3741  (2.7) | <0.001 | 3790  (9.3) | 4832  (3.5) | <0.001 | 1976  (4.3) | 6845  (4.9) | <0.001 | 513  (1.0) | 7746  (5.6) | <0.001 |
| All numbers are reported as absolute numbers and percentage if not stated otherwise. IQR: Interquartile Range; SD: Standard Deviation;  *: level 1: ≤ 500 Swiss francs (CHF); level 2: 501 – 1500 CHF: level 3: 1501 – 2500 CHF  **: Based on positive Pharmaceutical Cost Group (PCG). | | | | | | | | | | | | |

| Supplemental table 5: Comparison of Baseline characteristics between first time vaccinated in 2020/2021 patients (FTVP) and other patients (cohort). | | | | | |
| --- | --- | --- | --- | --- | --- |
|  |  | Overall | Subgroups | |  |
| Population |  |  | First time vaccinated 2020/2021 | Other | p |
| Number of cohort patients |  | 138,526 | 13,763 | 124,763 |  |
| Age ≥ 65 |  | 115,119 (83.1) | 11,698 (85.0) | 103,421 (82.9) | <0.001 |
| Swiss Nationality |  | 103,426 (74.7) | 9957 (72.3) | 93,469 (74.9) | <0.001 |
| Language in residence area | abroad | 931 (0.7) | 60 (0.4) | 871 (0.7) | <0.001 |
|  | French | 82,118 (59.3) | 8589 (62.4) | 73,529 (58.9) |  |
|  | German | 50,003 (36.1) | 4332 (31.5) | 45,671 (36.6) |  |
|  | Italian | 5474 (4.0) | 782 (5.7) | 4692 (3.8) |  |
| Sex | Female | 71,037 (51.3) | 7000 (50.9) | 64,037 (51.3) | 0.304 |
| At least one comorbidity* |  | 63,959 (46.2) | 6695 (48.6) | 57,264 (45.9) | <0.001 |
| Risk group | comorbidity + age | 40,552 (29.3) | 4630 (33.6) | 35,922 (28.8) | <0.001 |
|  | only age | 74,567 (53.8) | 7068 (51.4) | 67,499 (54.1) |  |
|  | only comorbidity | 23,407 (16.9) | 2065 (15.0) | 21,342 (17.1) |  |
| Deductible level** | Level 1 | 116,903 (84.4) | 12,133 (88.2) | 104,770 (84.0) | <0.001 |
|  | Level 2 | 12,329 (8.9) | 1052 (7.6) | 11,277 (9.0) |  |
|  | Level 3 | 9294 (6.7) | 578 (4.2) | 8716 (7.0) |  |
| Insurance model | Free-choice | 62,962 (45.5) | 6403 (46.5) | 56,559 (45.3) | <0.001 |
|  | Network | 13,230 (9.6) | 1214 (8.8) | 12,016 (9.6) |  |
|  | Family medicine | 41,112 (29.7) | 4251 (30.9) | 36,861 (29.5) |  |
|  | Telemedicine | 21,222 (15.3) | 1895 (13.8) | 19,327 (15.5) |  |
| Number of GP consultations,  median [IQR] |  | 3 [0, 6] | 4 [2, 7] | 3 [0, 6] | <0.001 |
| Number of specialist consultations, median [IQR] |  | 3 [1, 7] | 4 [1, 9] | 3 [1, 7] | <0.001 |
| Number of comorbidities,  mean (SD) *** |  | 1.52 (1.32) | 1.69 (1.29) | 1.51 (1.32) | <0.001 |
| Lung diseases |  | 10,687 (7.7) | 1066 (7.7) | 9621 (7.7) | 0.901 |
| Cardiovascular diseases |  | 10,925 (7.9) | 1248 (9.1) | 9677 (7.8) | <0.001 |
| Diabetes |  | 26,144 (18.9) | 2686 (19.5) | 23,458 (18.8) | 0.043 |
| Drug immunosuppression & autoimmune disease |  | 15,158 (10.9) | 1713 (12.4) | 13,445 (10.8) | <0.001 |
| Transplantations |  | 748 (0.5) | 80 (0.6) | 668 (0.5) | 0.525 |
| Cancer |  | 5330 (3.8) | 663 (4.8) | 4667 (3.7) | <0.001 |
| HIV**** |  | 1706 (1.2) | 120 (0.9) | 1586 (1.3) | <0.001 |
| Cerebral diseases |  | 2723 (2.0) | 250 (1.8) | 2473 (2.0) | 0.195 |
| Renal insufficiency |  | 455 (0.3) | 66 (0.5) | 389 (0.3) | 0.001 |
| Liver diseases |  | 136 (0.1) | 16 (0.1) | 120 (0.1) | 0.569 |
| Lymphoma, leukaemia or myeloma |  | 165 (0.1) | 22 (0.2) | 143 (0.1) | 0.184 |
| Living in nursing home |  | 6845 (4.9) | 768 (5.6) | 6077 (4.9) | <0.001 |
| All numbers are reported as absolute numbers and percentage if not stated otherwise.  *: At least one chronic disease from: heart, liver, lung, cerebral and kidney diseases, diabetes mellitus or immunocompromised patients with autoimmune diseases, drug immunosuppression, human immunodeficiency viruses (HIV), cancer, lymphoma, leukemia or myeloma or transplantations.  **: level 1: ≤ 500 Swiss francs (CHF); level 2: 501 – 1500 CHF: level 3: 1501 – 2500 CHF  ***: Based on positive Pharmaceutical Cost Group (PCG)  ****: Human immunodeficiency viruses  SD: Standard deviation. IQR: Interquartile Range. GP: General practitioner | | | | | |
